# Reimbursement decisions for medical services in Austria: an analysis of influencing factors for the hospital individual services catalogue between 2008 and 2020

**DOI:** 10.1186/s12913-022-07531-3

**Published:** 2022-02-15

**Authors:** Gregor Goetz, Dimitra Panteli, Reinhard Busse, Claudia Wild

**Affiliations:** 1 HTA Austria - Austrian Institute for Health Technology Assessment GmbH, Garnisongasse 7/20, 1090 Vienna, Austria; 2 Department of Health Care Management, Technische Universität Berlin, Strasse des 17. Juni 135 (H80), 10623 Berlin, Germany; 3 European Observatory On Health Systems and Policies, Brussels, Belgium

**Keywords:** Coverage, Reimbursement, Benefit catalogue

## Abstract

**Objectives:**

To (1) describe the (evidence-based) reimbursement process of hospital individual services, (2) evaluate the accordance between evidence-based recommendations and reimbursement decision of individual services and (3) elaborate potential aspects that play a role in the decision-making process in Austria.

**Method:**

The reimbursement process is described based on selected relevant sources such as official documents. Evidence-based recommendations and subsequent reimbursement decisions for the annual maintenance of the hospital individual service catalogue in Austria between 2008 and 2020 were analysed using a mixed methods approach, encompassing descriptive statistics and a focus group with Austrian decision makers.

**Results:**

118 evidence-based recommendations were analysed. There were 93 (78.8%) negative and 25 (21.2%) positive evidence-based recommendations. In total, 107 out of 118 evidence-based recommendations (90.1%) did not lead to a deviating reimbursement decision. We identified six aspects that may have played a role in the decision-making process for the annual maintenance of the hospital individual service catalogue, with clinical evidence being the most notable. Further aspects included quality assurance/organisational aspects (i.e., structural quality assurance), costs (if comparable to already existing medical services, not: cost-effectiveness), procedural aspects (e.g., if certain criteria for adoption have not been met formally through the proposals), “other countries” (i.e., taking into account how other countries decided) and situational aspects (such as the COVID-19 pandemic).

**Conclusions:**

There is good accordance between evidence-based recommendations and reimbursement decisions regarding hospital individual services in Austria. Beyond clinical evidence, organisational aspects seem to be considered often with regard to quality assurance but costs do not appear to play a major role. The Austrian system has mechanisms in place that can restrict widespread adoption of novel hospital individual services with uncertain clinical benefits. Future studies could investigate how well these mechanisms work and how they compare to other health systems in Europe.

**Supplementary Information:**

The online version contains supplementary material available at 10.1186/s12913-022-07531-3.

## Introduction

Coverage decision making is challenging because of concerns with incremental novelty of new interventions, their ever rising costs as well as a significant increase in expectations from patients [1]. With an arguably inflationary expansion of available health services but not necessarily a proportionate health outcome [2], it is increasingly important to assess the rational basis – the scientific evidence with regard to the benefits and harms – of medical interventions along the whole life cycle. The challenges in coverage decisions on new medical services utilising medical devices have long been exacerbated by comparably less strict European Union (EU) regulation when compared to drugs. Approval is generally faster and there is subsequently potentially an increased risk of harms for patients [3]. Even high-risk medical devices were often approved without the support of high-quality clinical evidence; in a comparative analysis of clinical evidence for market authorisation in Europe and the USA, it was revealed that approval in Europe was generally 3–5 years faster and subsequently associated with increased risk of harms for patients [3–5]. Many safety incidents and the criticism of the lax regulation led to a new EU regulation on medical devices (EU-MDR 2017/745), to be implemented in 2021 [6, 7]. Yet, it remains to be proven, if regulation alone can ensure safety/efficacy of novel interventions and lead to better accountability and transparency [8, 9].

Decision makers’ interest in health technology assessment (HTA) as a tool to support decisions by summarising best available evidence [10] has grown over the past decades [11]. Countries have increasingly established systematic approaches to assess the clinical evidence on medical services before their widespread adoption into the healthcare system. However, the linear adoption of such intervention (without, for instance, re-evaluations through the whole life cycle) can be associated with numerous risks [12]. These include, for instance, the introduction of medical interventions with doubtful value, the introduction of innovations that are isolated from ‘true’ health care needs, generating unnecessary variability in practice, maintaining no-added value care/ medical practices, and disregarding the current state-of-the art knowledge in clinical practice [12].

Consequently, new concepts such as early scientific advice, early awareness systems, as well as post-launch observation, re-assessment and potential disinvestment have increasingly been implemented [12]. In Austria, the introduction of new medical services and medical devices into the hospital individual services catalogue, as well as the amendment or deletion of already included services, has been supported by comparative evaluations of the clinical evidence since 2008. These evaluations take the form of evidence syntheses of comparative effectiveness and safety conducted by the by the Austrian Institute for Health Technology Assessment (AIHTA; Former: Ludwig Boltzmann Institute for HTA) [13]. An interdisciplinary steering committee coordinated by the Ministry of Health is responsible for the maintenance of the hospital individual services catalogue and uses the evidence syntheses for an annual update of the catalogue, including new interventions, but also excluding (disinvesting) interventions of uncertain clinical value [14, 15]. Such HTA reports can also be commissioned if a decision of disinvestment needs to be taken; e.g. for reimbursed hospital individual services with increased uncertainty around their clinical value [13, 15–17].

This system has been described and partly evaluated in earlier publications, concluding that clinical evidence is being well considered in decisions on in-/exclusion of services in the hospital catalogue, but proposing that other factors seem to influence the final decisions as well [13, 16, 17]. It is still not sufficiently clear which re-imbursement decisions have been exclusively supported by clinical evidence and which further factors influenced the decisions besides clinical evidence. This study is aims to provide a detailed description of the reimbursement process of medical services for the maintenance of the Austrian hospital individual services catalogue, taking the perspective of the decision makers, and to identify the additional factors that play a pivotal role in this decision-making process. Based on these aims, we formulated three research questions:

1. What are the specifics of the decision-making process for the reimbursement of novel hospital medical services in Austria beyond what has already been described in international literature?
2. In how far were reimbursement decisions of hospital individual services based on clinical evidence?
3. What other factors are taken into account in the decision-making process that may lead to deviations from the evidence-based recommendation in Austria?

## Methods

To describe the reimbursement process of hospital individual services in Austria, we analysed official documents of the Ministry of Health (MoH) and further manually searched for literature describing the Austrian health system in detail. Content from the focus group (see below) was used to validate and enrich this information.

The recommendations from all evidence syntheses of (new) medical services from 2008 to 2020 were analysed and contrasted to the reimbursement decisions for the annual update of the Austrian hospital individual services catalogue. A mixed methods approach was applied:

As the first step, the existing dataset including all evidence syntheses and their evidence-based recommendations and reimbursement decisions until 2016 [16] was inspected and verified, then the dataset was updated until 2020 using the annual hospital individual services catalogues.

Subsequently a comparison of recommendations and subsequent reimbursement decisions applying descriptive statistical methods was conducted to calculate the accordance between these two variables. For this purpose, both variables were dichotomised accordingly: recommendations for unconditional reimbursement and reimbursement with restriction were categorised as positive, while negative recommendations covered both preliminary rejections and full rejections. Similarly, the reimbursement decision was positive if the technology was funded and, thus, included both unconditional reimbursement and reimbursement with restrictions (e.g., tied to certain conditions and requiring pre-approval). Negative reimbursement decisions covered, on the contrary, no inclusion in the hospital individual services catalogue and inclusion solely for the purpose of documentation (i.e., through so-called “XN codes”, see below). To calculate the accordance between these variables, a contingency table was created using MS Excel.

In the second step, a focus group was conducted with Austrian decision makers to identify further aspects that may influence the reimbursement of hospital individual services. Evidence-based recommendations for which the reimbursement decision deviated were identified. On the basis of these deviations, questions for the focus group were prepared. We contacted a convenience sample of members (*n* = 4) of the expert committee updating the hospital individual services catalogue per e-mail. Our criterion for participation was at least one year of active experience on the committee.

We conducted and recorded the focus group via Zoom and used a semantic-content based transcription system according to Kuckartz [18, 19]. We applied a qualitative content analysis in Atlas.ti, using the structural content analysis method according to Mayring [20]. We first created a system of categories (“aspects”) and defined these according to the domains specified in the EUnetHTA Core Model [21]. After a first round of analysis, we added inductively determined categories to strengthen the category system and analysed the content accordingly one more time. The system of categories can be found in Table 1. One person (GG) was involved in coding relevant text passages and creating new categories, and another person (DP) verified the category tree and coding. After the analysis, relevant quotes for this publication were translated to English.

**Table 1:** Category system created for the focus group analysis and n of codes, n of coded citations within the qualitative content analysis

Aspect (Code-Group)	Inductive or deductive	Source	Description	N of codes	N of coded citations in focus group
Clinical evidence (EFF/SAF)	Deductive	[21]	This aspect covers whether a new hospital medical service is more effective (EFF) and at least as safe (SAF) as the comparator	1	1
Costs	Deductive and inductive	[21]	This domain was broad and covers not only comparative economic evaluations as defined in the EUnetHTA Core Model, but also whether the cost of the new EMS played some sort of a role in the decision making process	1	1
ORG/ quality assurance	Deductive and inductive	[21]	“The domain of **Organisational Aspects** (ORG) considers the ways in which different kinds of resources (e.g. material artefacts, human skills and knowledge, money, attitudes, work culture) need to be mobilised and organised when implementing a technology, and the consequences they may further on produce in the organisation and the health care system as a whole. Organisational issues include e.g. work processes and patient/participant flow, quality and sustainability assurance, centralisation, communication and co-operation, managerial structure, and acceptance of a technology.” Within this domain, especial focus was attributed to quality assurance. We linked the broad term quality assurance with ORG after noting that there is considerable overlap	5	16
ETH/SOC	Deductive	[21]	“The **Ethical Analysis (ETH**) domain considers prevalent social and moral norms and values relevant to the technology in question. It involves an understanding of the consequences of implementing or not implementing a healthcare technology in two respects: with regard to the prevailing societal values and with regard to the norms and values that the technology itself constructs when it is put into use “ “The Patients and **Social Aspects (SOC)** domain takes patients or individuals in whose care a health technology is used as a point of reference in an HTA. Patients Aspects relate to issues relevant to patients, individuals and caregivers. Patient refers to a person who receives (or has received) and uses (or used) health technologies and health services in the healthcare sector. The term individual is sometimes used synonymously with ‘patient’, but it can also refer to a healthy individual, who receives health technologies, e.g. a person taking part in a screening programme. The term caregivers (sometimes referred to as carers) refers to family, friends and other persons from the patient’s/individual’s social network, who provide care to the patient and are in other ways involved during the course of the disease. It excludes those paid to give care, such as healthcare professionals. Social Aspects are related to social groups, that is specific groupings of patients or individuals that may be of specific interest in an HTA, such as older people, people living in remote communities, people with learning disabilities, ethnic minorities, immigrants etc.”	1	1
REG	Deductive	[21]	**Regulatory aspects (REG)** are covered within the TEC/ description and technical characteristics of the technology EUnetHTA domain. The regulatory (REG) status addresses primarily whether (and for which indications) the technology received a marketing authorisation/ CE marking	0	0
Situational Aspect	Inductive	Self-defined	These aspects were defined as a set of (external) circumstances that may have influenced a reimbursement decision at a given time	1	4
Other countries	Inductive	Self-defined	The aspect “other countries” covers the potential influence of prior reimbursement coverage decisions of certain technologies from other countries	2	3
LEG	Deductive	[21]	“The objective of the **Legal Aspects (LEG)** domain is to assist the HTA doers in detecting rules and regulations which need to be taken into consideration when evaluating the implications and consequences of implementing a health technology. Rules and regulations have been established to protect the patient’s rights and societal interests. The rules and regulations may be a part of patient rights legislation, data protection legislation, or health care personnel’s provisions, rights and duties in general (…)”	0	0
Procedural aspects	Inductive	[15]	Procedural aspects are linked to the criteria used within the general reimbursement process of the LKF Model	9	14

## Results

### Characteristics of focus group respondents

Three experts accepted the invitation: one of the experts was from the MoH (responsible for the maintenance of the hospital individual service catalogue) and the remaining two experts were from regional health funds responsible for financing of hospitals. More detailed information on the focus group respondents can be found in Table 2.

**Table 2:** Characteristics of focus group respondents

Number	Committee experience, in years	Expertise/ current work	Professional background and Qualifications
1	20	Medical documentation, Health Planning at Ministry of Health in Austria	Medical Doctor (MD)
2	10	Health Planning, Health Data & Medical Quality Management Officer at a regional Health Fund of Austria	Medical Doctor (MD)
3	1	Health Planning, Control & Quality at a Regional Health Fund of Austria	Medical Doctor (MD)

*MoH* Ministry of Health

### General reimbursement process of hospital individual services

Starting in 1997, Austria implemented gradually an activity-based hospital payment system called “Leistungsorientierte Krankenanstaltenfinanzierung (LKF)”. The aim of this diagnostic-related groups (DRG) system is – as in other countries – to increase transparency and efficiency of hospitals and accordingly to contain costs. The LKF system relies on two pillars: the core area (nationally uniform for all hospitals) and the steering area (with criteria to adapt to the level of hospital care and to Federal States) [22].

Similar to classic DRG systems, the relative costliness of individual cases [15, 22] is determined based on regular calculations using data from reference hospitals on costs of average cases. The diagnosis-related “cases” form groups (so-called “leistungsorientierte Diagnosefallgruppen”/LDF), which are either procedure-related or diagnosis-related. Based on LDF groups, point-value scores are calculated and determine the reimbursement to individual hospitals [15, 22].

**Fig. 1:**
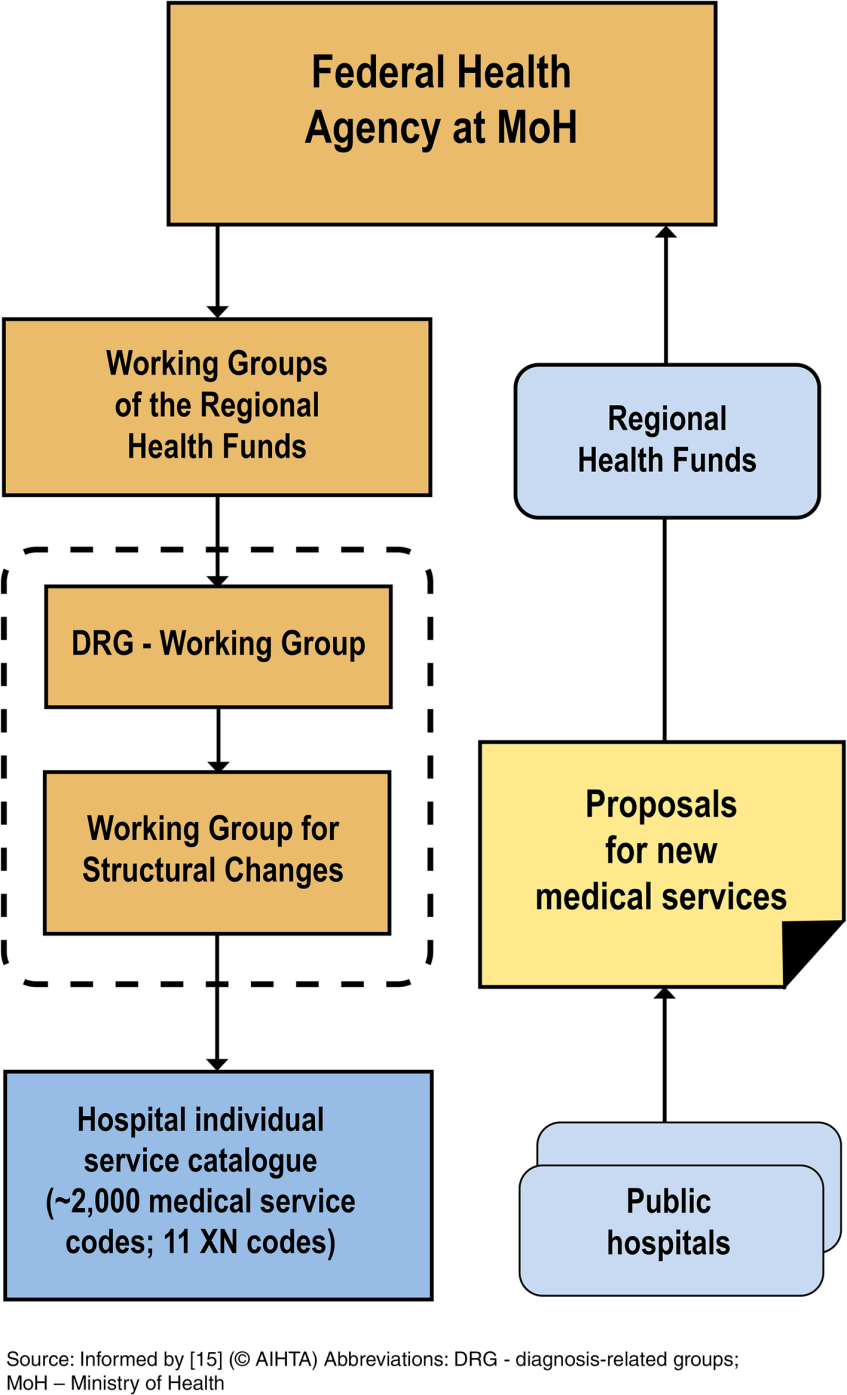
Process of hospital individual service adoption in Austria. Source: Informed by [15] (© AIHTA) Abbreviations: DRG - diagnosis-related groups; MoH – Ministry of Health

The LKF model is annually updated by an interdisciplinary working group (hospital committee) coordinated by the MoH. In this process, new – most often costly – medical services can be introduced to the “individual services catalogue” – a positive list of around 2,000 medical services – or price changes can be accounted for [15, 22]. Public hospitals can submit proposals on which medical services should be considered for inclusion into the hospital individual services catalogue (see Fig. 1).

There are key criteria for adoption of novel, or differentiation of already existing, medical services. A hospital individual service that is newly developed, technically established with a clear medical indication and with adequate scientific evidence, with certain economic relevance and a unit of service that is distinguishable from other services would, for instance, fulfil the criteria for inclusion into the hospital individual services catalogue. A deletion of a service may be considered, if, for instance, the service does not have sufficient evidence supporting its use or is no longer provided/ does not follow current medical standards [15]. The full list of all in- and exclusion criteria as well as criteria for deletion (disinvestment) can be found in Table 3.

**Table 3:** Criteria for inclusion, exclusion and deletion of a hospital medical service used by the steering committee of the hospital individual services catalogue

**Criteria for adoption** of a novel, or differentiation of an already existing, hospital medical service	•The service is newly developed or required to reflect medical advances •The service is technically established •Adequate scientific evidence is available •There is a strictly defined medical indication for the service •The service and unit of service is clearly defined and distinguishable from other service items •The service is of economic relevance (cost, frequency)
**Exclusion criteria** regarding a potential adoption of a hospital medical service	•Incomplete proposals •Examinations and treatments that are typical and recurring components of various diagnostic or therapeutic medical service •Services that are included in existing case rates •Different surgical techniques for the same medical services •Medications, except in the area of oncology •Service items that contain manufacturer-related drug, device, or other manufacturer-related material claims
**Criteria for deleting** (disinvesting from) a hospital medical service	•The service is no longer provided •The service no longer meets current medical standards •The service does not have sufficient evidence •The service is not relevant for any of the topics: billing, observation, planning, control, quality assurance •The service recording is highly incomplete and not valid and the data quality cannot be easily improved

Source: [15]

The focus group respondents described the hospital individual services catalogue as a rather broadly defined catalogue containing medical services that are eligible for financing. The respondents described the evolution of the individual services catalogue from capturing all services that are reimbursed in Austrian hospitals (note: with certain economic relevance), in the early years of implementing the DRG-system to additional aspects of relevance such as quality assurance and planning of services covered.

“(…) This means that a certain economic relevance is required to be considered/ not considered to be depicted in this catalogue. Small, less costly and less frequently utilised benefits can be coded with equivalent codes without being depicted in the catalogue as such. Capturing these benefits that are financed in Austrian hospitals was the initial idea of the hospital individual services catalogue. Later, other aspects such as quality assurance and planning were introduced as further relevant aspects of the catalogue. Hence, a more differentiated presentation began to evolve, without having a direct impact on financing due to the fact that the latter rates are still in the background (for which the more detailed services are summarised) (…)” (Focus group response 2020, 0:12:17).

### Evidence-based reimbursement

Since 2008, interventions that are being considered for introduction into or deletion from the hospital individual services catalogue undergo evaluations regarding their comparative effectiveness and safety. To prioritise relevant topics for these assessments, proposals by public hospitals and their regional hospital federations are listed and prioritised according to potential for true innovation, high cost and high volume of patient population, risk or uncertainty regarding value [15]. For these prioritised topics, evidence syntheses are conducted and recommendations based on GRADE [23] are formulated and provided to the MoH [14].

Informed by both published literature [16] and the focus group response, evidence-based recommendations and their impact on or implication for reimbursement decisions can be described as follows. Evidence-based recommendations in favour of the intervention (*recommended*) are usually formulated when there is strong evidence indicating a clinical benefit of the adoption of the intervention, and they support an adoption as a reimbursable hospital individual service without restrictions. If the clinical evidence is less robust, but still points in a beneficial direction, an *adoption with restrictions* is recommended. In those cases, decision makers will usually include the intervention as a fully reimbursable hospital individual service, but may couple it with certain restrictions (e.g., only in some hospitals with certain qualifications) and re-evaluate it at a later stage, potentially linked to the completion of registered, ongoing randomised clinical trials (RCTs). A *preliminary rejection* is usually recommended when there is no or inconclusive evidence, indicating a clinical benefit. In this scenario, the decision makers do not reimburse the intervention as such. However, it still may be included in the hospital individual services catalogue as a new examination and treatment method for the purpose of documentation (with a XN-Code). In 2021, overall eleven medical services are XN coded for documentation only. Then, the amount of eligible reimbursement is usually not changed; rather, hospitals usually get the minimum reimbursement based on LKF codes that were used prior to submitting the proposal. Update assessments are then conducted leading to either a) an inclusion as a reimbursable benefit, b) prolongation of the preliminary status, or c) the non-inclusion of medical service, i.e. its preliminary status will be deleted [15]. A recommendation *to reject* a certain intervention is formulated if there is strong evidence indicating no clinical benefit or if the harms exceed the benefits, leading to no inclusion in the hospital individual services catalogue. Table 4 gives a broad overview of evidence-based recommendation categories and the ideal type of responses to the recommendations.

**Table 4:** Categories of evidence-based recommendations and ideal types of subsequent decisions of the decision makers

Category	Description of recommendation	Decision maker’s response
Recommended	Strong evidence indicating an additional benefit of using the intervention	Inclusion as an unconditionally reimbursable hospital medical service
Recommended with restriction	Less robust evidence indicating an additional benefit of using the intervention Ongoing studies may have a considerable influence on the effect estimate, leading to the necessity to re-evaluate the medical service at a later stage	Decision makers reimburse the medical service with restriction: Services require approval and can be linked to certain conditions (e.g. university hospitals, cardiology centres etc.). These services are usually re-evaluated at a later stage
Preliminary rejected	No evidence or inconclusive evidence available to assess an additional benefit of the intervention of interest	No standard reimbursement. It may still be included in the hospital individual services catalogue as a new examination and treatment method (“Neue Untersuchungs- und Behandlungsmethode/ NUB”; XN-Codes) for the purpose of documentation. A re-evaluation takes place on the basis of the evidence-based recommendation
Rejected	Strong evidence indicating no benefit and/ or harm of the intervention	No inclusion

Source: Informed by [16], the general description of the maintenance of the hospital individual services catalogue [15] and the focus group response

### Quantitative analysis of accordance between evidence-based recommendation and reimbursement decision

The quantitative comparison between evidence-based recommendation and subsequent decision for the annual maintenance of the hospital individual services catalogue in Austria shows a high rate of accordance. Between 2008 and 2020, 107 out of 118 (90.7%) evidence-based recommendations received an aligned decision (based on clinical evidence). There were only four negative recommendations (3.4%) for medical services which were then nevertheless adopted or not removed from the individual services catalogue. Further, there were seven positive evidence-based recommendations (5.9%) that resulted in a negative reimbursement decision. Table 5 gives a broad overview of the accordance between evidence-based recommendation and reimbursement decision. The whole list of all deviations (incl. product names) can be found in the online Additional file 1.

**Table 5:** Cross table of evidence-based recommendations and subsequent decisions of the annual maintenance of the hospital individual services catalogue in Austria

Reimbursement decision	Evidence-based recommendation		
	**Negative**	**Positive**	**Total**
Negative	89 (75.4%)	7 (5.9%)	96 (81.4%)
Positive	4 (3.4%)	18 (15.3%)	22 (18.6%)
Total	93 (78.8%)	25 (21.2%)	118 (100%)

It is to be noted that all deviations that occurred were not “strong” deviations. All of the “positive” recommendations that received a negative reimbursement decision were “reimburse with restriction” recommendations (as opposed to a strong recommendation for inclusion). Similarly, most of the deviations in the other direction (negative recommendation followed by a positive reimbursement decision) received a “preliminary” rejection and not a “full rejection”.

### Aspects influencing reimbursement decisions

Based on the focus group and descriptive statistics/ contingency table of the accordance, we identified six types of aspects that may have played a role in the decision-making process for the annual maintenance of the hospital individual services catalogue. An overview of our findings can be found in Table 6.

**Table 6:** Summary of findings regarding aspects taken into consideration into the decision making process

Aspects	Findings
*Clinical evidence:*	Decision makers noted that this was the most essential aspect, as also shown in the accordance between evidence-based recommendation and reimbursement decision.
*Quality assurance/ organisational aspects*:	Structural quality assurance through setting conditions for the use of a medical service (provider’s capacity, infrastructure and processes to provide the specific care in high-quality). These are often linked to organisational aspects.
*Costs*	If costs are comparable to already existing medical services, the new medical service may not be included in the catalogue separately.
*Procedural aspects*	These aspects are linked to the general criteria for adoption of novel hospital medical service (e.g., incomplete proposal may lead not to include a hospital service).
*Other countries*	How other countries (especially Germany) decided on the potential adoption of a certain medical service may have also influenced some reimbursement decisions.
Situational aspects	The COVID-19 pandemic was identified as a situational aspect hindering a reimbursement of medical services.

Notes: Three HTA-related aspects from the deductive coding categories were either not mentioned or not applicable: *ethical and social aspects, regulatory aspects, and legal aspects*

Comparative clinical effectiveness and safety (***clinical evidence***) notably constitute the major aspect in the decision-making process for the reimbursement of hospital medical services in Austria. This was evident both in the accordance between evidence-based recommendations and subsequent reimbursement decisions so far (see Table 5) and during the focus group. The general principles of the annual maintenance of the hospital individual services catalogue were described as follows:

*“(…) Or something comes along where you’d say: Oho! That's something scientifically new. We haven't had that yet, so that's a new variation for the therapy of (e.g.) hypotension by implanting something (…). Then, of course, it is interesting to look at the clinical benefit. And then there’s the question: Does it make sense or not? Then it's a matter of evaluating the evidence: Are there any studies at all, and if there are any, then you look at the benefit (…).” (Focus group response 2020, 0:21:58)*

#### Quality assurance and organisational aspects

While distinct in the deductive category system, these aspects are not described separately due to significant overlap in the focus group responses. Notably, these are central aspects in the decision-making process to determine how medical services are included. This aspect evolved over time and the whole hospital individual services catalogue became, consequently, more detailed:

*“Quality assurance is another aspect that has been added and that has led (…) to procedures (…) being mapped in a more granular way than they have been in the catalogue up to now, without the flat rates per case being split in the background (…).” (Focus group response 2020, 0:17:25)*

Structural quality assurance plays a role in this context by setting conditions for the use of a medical service. If evidence is not that clear or other aspects hinder prompt decision making (such as situational aspects), the aspect of structural quality assurance was considered crucial before adopting a new treatment method into the standard financing system. The question arises which hospitals should be able to use a medical service so that it is assured that evidence-based services are delivered in hospitals only with the right qualifications and technical equipment. In one specific scenario of deviations the following was, among others, discussed:

*“Well, the quality assurance/ I can only approve at the hospital level. That means: I need some criterion (...). The hospital is allowed to do it now because they somehow (...) have a heart catheter or no, they are not allowed to do it because they don't have heart surgery. So you have to/ that is still worth discussing.” (Focus group response 2020, 1:14:36)*

With regard to minimum volume standards, it was mentioned that these do not play a role in the decision-making process but it is still useful to be able to document them.

The adoption may further be coupled to some hospitals for which certain local specialisations are already existent. The interviewer framed as to whether the discussants saw that local specialisations and the structural quality dimension also needs to be seen in the organisational context, which they agreed upon.

#### Costs

The aspect of whether a therapy is cost-effective does not appear to play a role in the decision-making process per se. However, if costs are comparable to already existing medical services, the new medical service may not be included in the catalogue separately.

*“(…) It may also be that it turned out that the cost that was sent along for the LDL apheresis was the same as the plasmapheresis. There is also a basic service in the catalogue for selective cell adsorption. And then for both (…): Continue to code them under this mapped basic individual service.” (focus group response 2020, 0:55:07)*

#### Procedural aspects

These aspects are linked to the general criteria for adoption of hospital individual services. That is, in certain contexts, a hospital individual service was not included as a reimbursable hospital individual service if, for instance, the proposals were incomplete (see exclusion criteria for medical services, above). Additionally, when annual maintenances were first initiated, there was no system in place to delete a hospital individual service. Some interventions were evaluated but could not be deleted. The system to delete a hospital individual service was established at a later stage.

#### Other countries

The focus group mentioned being influenced by how other countries (especially Germany) decided on the potential adoption of a certain medical service:

*“(…) In Germany, as far as I know, it is also the case that it is not part of the standard funding. At least it wasn't in the spring of this year. They said they were waiting for another large study and when that was finished, 2021, then a decision would be made. So that's how it was written (…) at the back (note in the report).” (focus group response 2020, 1:11:01)*

### Situational aspects

The COVID-19 pandemic was identified as a situational aspect hindering the reimbursement of medical services. The COVID-19 pandemic hindered a physical meeting for two medical services that received a positive recommendation. In both of these cases, the reimbursement decision was negative, although the medical services were included in the hospital individual services catalogue for the purpose of documentation. With respect to the question as to why the reimbursement decision was not positive, the focus group responded that the cases.

*“(…) were completed during the first lockdown and are now both included as a NUB [note: new examination and treatment method] (…). Had we had a videoconference or a meeting at that time, we would have discussed/ do we decide for a service that requires authorisation [*=*restricted reimbursement], and take it into the standard funding or do we leave it as a NUB. We would have done one of the two variants in any case. We have given ourselves the minimum variant here, so to speak, (…). I cannot say how this would have turned out if we had discussed it. It's possible that we would now have two more services requiring authorisation.” (focus group response 2020, 1:08:38)*

### Reproducibility of reimbursement decisions

Focus group participants recalled a specific scenario, in which they believed that they may have been *too strict* in excluding a medical service, which should have been partly reimbursed followed by close monitoring and re-evaluation at a later stage. However, in this specific scenario the focus group did not remember the reasons behind the decision, as it had taken place over ten years earlier.

## Discussion

118 evidence-based recommendations were analysed. There were 93 (78.8%) negative and 25 (21.2%) positive evidence-based recommendations. In total, 107 out of 118 evidence-based recommendations (90.1%) did not lead to a deviating reimbursement decision. We identified six aspects that may have played a role in the decision-making process for the annual maintenance of the hospital individual services catalogue. The clinical evidence was the most notable aspect. Further aspects included quality assurance/organisational aspects service, costs (if comparable to already existing codes of a medical service, not: cost-effectiveness), situational aspects (such as the COVID-19 pandemic), procedural aspects (e.g., if certain criteria for adoption have not been met formally through the proposals) and “other countries” (i.e., taking into account how other countries decided).

The results of this analysis complement older analyses of the evidence-based reimbursement process of hospital medical services in Austria. A descriptive analysis [13] regarding reimbursement processes in Austria (early assessment for coverage decisions) found 95 HTA assessments between 2008 and 2018. Their unit of analysis were HTA assessments (instead of recommendations as chosen in our analysis). In the majority of the cases, the evidence-based recommendations translated directly into reimbursement decisions. Yet, the analysis also showed that while 15 (21.7%) hospital medical services were recommended by the conducted HTA reports, 25 (36.2%) hospital medical services were finally included in the hospital individual services catalogue (either fully or with restrictions). The study authors found that there is an interdependency of coverage decisions and existing evidence. Our quantitative analysis complements this finding by calculating the accordance directly through the cross table in which both of these variables (recommendation and decision) are dichotomised, and confirms it for this broader sample of evidence-based recommendations.

Surprisingly, the quality of the evidence and the risk classification of a medical device were not specifically mentioned in the focus group in our study. However, it must be noted that the quality of the evidence indirectly plays a role (through the role of evidence as such). This is somewhat in contrast to a retrospective analysis of 78 medical device syntheses (time period: 2008–2015) [16] that tried to identify factors that impact coverage decisions in Austria [16]. In this analysis, it was found that despite low clinical evidence, some high-risk devices (for only a few patients) received a positive decision. Logistic regression analysis showed that there was no significant association between variables addressing the quality of the evidence and reimbursement decision for risk class III devices. High-risk device characteristics were found to be positive predictors for reimbursement within the group of medical devices for which no RCTs were available. However, for class II devices, variables addressing the quality of the evidence were positive predictors for the reimbursement decision [16].

Although there are clearly mechanisms in place in Austria that hinder widespread adoption of promising novel technologies without clear clinical evidence, transparency within the decision-making process could be further enhanced. A health system should be explicit about the resource allocation process [24]. There are broadly three conditions that should be met in order to ensure accountability: publicity, relevance and revisability [24]. In the decision-making process of hospital medical services in Austria, both relevance (through an agreement of stakeholders) and revisability (through the system to delete already included hospital services and re-evaluations) are already fulfilled to some degree. It appears that publicity could further be strengthened.

More specifically, the steering committee could make the reasons of their decisions public. This could foster a deliberative process [25] that not only increases accountability of the decisions but could also engage a discussion as to whether existing criteria are sufficient or new criteria (such as cost-effectiveness) could or should be incorporated into the decision-making algorithm. If adaptations on the decision-making algorithm are to be considered, one could further conduct a scientific comparative health system analysis and engage the public through patient involvement and a public consultation. This would also ensure that patients and their organisations would have a stronger role in the decision-making process.

To increase accountability and encourage a stringent use of transparent criteria based on specific values, multi-criteria decision analysis (MCDA) could further be a tool considered for the Austrian context. MCDAs can not only inform funding decisions based on diverse criteria, but can further help to synthesise complex value trade-offs to support priority-setting decisions in a healthcare system using a rational and transparent approach [26, 27]. In the Austrian context, the aspects that are already considered besides clinical evidence as illustrated in our analysis could be incorporated transparently in such an MCDA and additional aspects (such as costs) could be considered.

### Limitations

The results of this analysis should be interpreted in light of its limitations: Firstly, in the calculation of the accordance between evidence-based recommendation and reimbursement decision there was sometimes not a clear cut-off between existing categories. However, we still believe that the dichotomisation of both variables is useful for capturing the accordance between recommendations and decisions quantitatively.

Secondly, we did not conduct regression analysis or logistic analysis as done in a previous study [16] that tried to capture factors influencing reimbursement decisions. We did not attempt to show causality between these two variables. However, since factors influencing reimbursement decisions have thus far only been investigated retrospectively, we believe that there are several limitations of conducting regression analysis for our defined research questions.

Thirdly, the reader must be aware that the results of the focus group are not generalisable to all steering committees. This analysis is limited to the annual maintenance of the hospital individual services catalogue in Austria that includes medical devices, outpatient services and some oncological drugs in the hospital [15]. Although the results may not be directly applicable to all settings due to variations in reimbursement processes and context across countries (e.g., health system, economic and social conditions), they are still useful for fostering a deeper understanding of both the annual process for maintaining the hospital individual services catalogue in Austria and the principles behind how deliberative processes in this area of decision making can be (further) developed.

## Conclusion

There is overall good accordance between evidence-based recommendations and reimbursement decisions of hospital medical services in Austria. While the aspects that played a role for specific decisions are difficult to ascertain, it is clear that they went beyond clinical evidence. While quality assurance and organisational aspects seem to be considered often, costs do not appear to play a major role. It appears that the Austrian system has mechanisms in place that can restrict widespread adoption of novel hospital medical services with uncertain clinical benefits. Future studies could investigate how well these mechanisms work and how they compare to other health systems in Europe.

## Supplementary Information

**Additional file 1.**

## Data Availability

The datasets generated and/or analysed during the current study are not publicly accessible but can be made available from the corresponding author upon reasonable request.
