# Supplementary material for: Reimbursement decisions for medical services in Austria: an analysis of influencing factors for the hospital individual services catalogue between 2008 and 2020
Source: BMC Health Serv Res. 2022 Feb 15;22:205. doi: 10.1186/s12913-022-07531-3 (PMC8848955; doi:10.1186/s12913-022-07531-3)
Supplement: Supplementary file 1 — Additional file 1. [file 12913_2022_7531_MOESM1_ESM.docx]

**Online Appendix 1: List of deviations**

The seven deviations (5.9%) that received a positive recommendation and negative decision were:

- Safyre^TM^ - adjustable sling for women with marked stress incontinence after conservative treatment options have been exhausted (year: 2008)
- Pro-ACT^TM^ - adjustable micro-balloon for men with marked stress incontinence after exhaustion of conservative treatment options (year: 2008)
- LDL apheresis in patients with severe familial hypercholesterolemia of homozygous and refractory heterozygous expression (year: 2008)
- Selective cell adsorption in inflammatory bowel disease (year: 2008)
- Selective Internal Radiotherapy (SIRT) for colorectal cancer metastasized to the liver using SIR-Spheres® (year: 2011)
- Percutaneous implantation of a probeless pacemaker (year: 2020)
- Implantation of a telemedical pulmonary artery pressure sensor (year: 2020)

There were some four deviations (3.4%), receiving a negative recommendation followed by a positive reimbursement decision:

- Percutaneous nucleolysis for the treatment of symptomatic disc herniation (year: 2009)
- Facet or sacroiliac joint injections (year: 2009)
- Intradiscal electrotherapy for the treatment of symptomatic disc hernia (year: 2009)
- Cervical prostheses for chronic pain or neurological deficits due to disc degeneration (year: 2010)
